# The amino acid transporter SLC36A4 regulates the amino acid pool in retinal pigmented epithelial cells and mediates the mechanistic target of rapamycin, complex 1 signaling

**DOI:** 10.1111/acel.12561

**Published:** 2017-01-13

**Authors:** Peng Shang, Mallika Valapala, Rhonda Grebe, Stacey Hose, Sayan Ghosh, Imran A. Bhutto, James T. Handa, Gerard A. Lutty, Lixia Lu, Jun Wan, Jiang Qian, Yuri Sergeev, Rosa Puertollano, J. Samuel Zigler, Guo‐Tong Xu, Debasish Sinha

**Affiliations:** ^1^ Department of Ophthalmology of Shanghai Tenth People's Hospital and Laboratory of Clinical Visual Science of Tongji Eye InstituteTongji University School of MedicineShanghaiChina; ^2^ The Wilmer Eye InstituteThe Johns Hopkins University School of MedicineBaltimoreMDUSA; ^3^ National Eye InstituteNational Institutes of HealthBethesdaMDUSA; ^4^ Cell Biology and Physiology CenterNational Heart, Lung and Blood InstituteNational Institutes of HealthBethesdaMDUSA; ^5^ Translational Medical Center for Stem Cell TherapyShanghai East HospitalTongji University School of MedicineShanghaiChina; ^6^ The Collaborative Innovation Center for Brain ScienceTongji UniversityShanghaiChina

**Keywords:** amino acid transporter (PAT4/SLC36A4), age‐related macular degeneration, coordinated lysosomal expression and regulation (CLEAR) network, lysosomes; mechanistic target of rapamycin, complex 1 (mTORC1), mouse model, retinal pigmented epithelium (RPE), photoreceptor degeneration, signal transduction, transcription factors EB (TFEB) and E3 (TFE3), visual cycle proteins

## Abstract

The dry (nonneovascular) form of age‐related macular degeneration (AMD), a leading cause of blindness in the elderly, has few, if any, treatment options at present. It is characterized by early accumulation of cellular waste products in the retinal pigmented epithelium (RPE); rejuvenating impaired lysosome function in RPE is a well‐justified target for treatment. It is now clear that amino acids and vacuolar‐type H^+^‐ATPase (V‐ATPase) regulate the mechanistic target of rapamycin, complex 1 (mTORC1) signaling in lysosomes. Here, we provide evidence for the first time that the amino acid transporter SLC36A4/proton‐dependent amino acid transporter (PAT4) regulates the amino acid pool in the lysosomes of RPE. In *Cryba1* (gene encoding βA3/A1‐crystallin) KO (knockout) mice, where PAT4 and amino acid levels are increased in the RPE, the transcription factors EB (TFEB) and E3 (TFE3) are retained in the cytoplasm, even after 24 h of fasting. Consequently, genes in the coordinated lysosomal expression and regulation (CLEAR) network are not activated, and lysosomal function remains low. As these mice age, expression of RPE65 and lecithin retinol acyltransferase (LRAT), two vital visual cycle proteins, decreases in the RPE. A defective visual cycle would possibly slow down the regeneration of new photoreceptor outer segments (POS). Further, photoreceptor degeneration also becomes obvious during aging, reminiscent of human dry AMD disease. Electron microscopy shows basal laminar deposits in Bruch's membrane, a hallmark of development of AMD. For dry AMD patients, targeting PAT4/V‐ATPase in the lysosomes of RPE cells may be an effective means of preventing or delaying disease progression.

## Introduction

Crystallins are highly abundant proteins of the lens, essential for maintaining its transparency and refractivity. In addition to their roles as structural elements in the lens, crystallins may also have diverse functions in other parts of the eye (Horwitz, 2003; Piatigorsky, 2008; Zigler & Sinha, 2015). βA3/A1‐crystallin, a member of the β‐crystallin subfamily encoded by the *Cryba1* gene, is also expressed in retinal pigmented epithelial (RPE) cells and astrocytes (Parthasarathy *et al*., 2011).

We have demonstrated that βA3/A1‐crystallin in the RPE is localized to the lysosomal lumen, where it regulates endolysosomal acidification by modulating V‐ATPase, thereby affecting lysosomal clearance by both phagocytosis and autophagy (Zigler *et al*., 2011; Valapala *et al*., 2014a,b). We have shown that βA3/A1‐crystallin binds to ATP6V_0_A1/V_0_‐ATPase and is involved in the mechanistic target of rapamycin, complex 1 (mTORC1) signaling pathway in RPE cells. Loss of βA3/A1‐crystallin results in decreased V‐ATPase activity, elevated lysosomal pH, activation of mTORC1, and inhibition of autophagy (Valapala *et al*., 2014a).

It is now recognized that an interplay between V‐ATPase and amino acids is essential in regulation of mTORC1 signaling (Zoncu *et al*., 2011). The proton‐assisted amino acid transporter (PAT)/solute‐linked carrier 36 (SLC36) family members regulate intracellular amino acid concentrations and mTORC1 signaling in lysosomes (Taylor, 2014). Recently, SLC38A9 (solute carrier family 38, member 9) was identified as an amino acid sensor that activates mTORC1 activity by interacting with the Ragulator‐Rag GTPase scaffolding complex in lysosomes (Rebsamen *et al*., 2015; Wang *et al*., 2015). Here, we show for the first time that PAT4/SLC36A4, a member of the PAT/SLC36 family, is expressed in RPE cells and is involved in the lysosomal dysfunction caused by loss of βA3/A1‐crystallin. PAT4 can mediate the amino acid‐sensing mechanism that regulates mTORC1 activation inside the cell (Heublein *et al*., 2010). It has been shown that Rab12 promotes constitutive degradation of PAT4 (Matsui & Fukuda, 2013). The accumulation of PAT4 in Rab12 knockdown cells increased mTORC1 activity and decreased autophagy.

mTORC1 signaling modulates lysosomal homeostasis (Laplante & Sabatini, 2013). As the RPE maintains the health of photoreceptors, preserving its normal clearance functions is essential to insure functional integrity of the neural retina (Strauss, 2005). Impaired lysosome‐mediated clearance results in toxic accumulation of undegraded waste products within the RPE, severely stressing these cells (Sinha *et al*., 2016). Further, the accumulated toxic material may be released by the RPE, thereby generating subretinal drusenoid deposits and drusen (deposits in Bruch's membrane), two signs indicative of development of dry age‐related macular degeneration (AMD), a major cause of vision loss in the elderly (Sivaprasad *et al*., 2016). With the human lifespan increasing, the management of aging‐related diseases becomes more important. There currently is no definitive treatment or prevention for dry AMD (Buschini *et al*., 2015). A new treatment that targets early dry AMD before significant vision loss would have great benefit for these patients.

We have now generated a global knockout mouse for *Cryba1*. These mice have pathological changes in the retina, mimicking some characteristics of dry AMD. Using this model, we provide novel evidence that the βA3/A1‐crystallin/PAT4/V‐ATPase complex is a potential therapeutic target for preventing or delaying the progression of dry AMD.

## Results

### βA3/A1‐crystallin interacts with PAT4, and loss of βA3/A1‐crystallin elevates cellular amino acid concentration in RPE cells and induces the mTORC1 pathway

**Figure 1:**
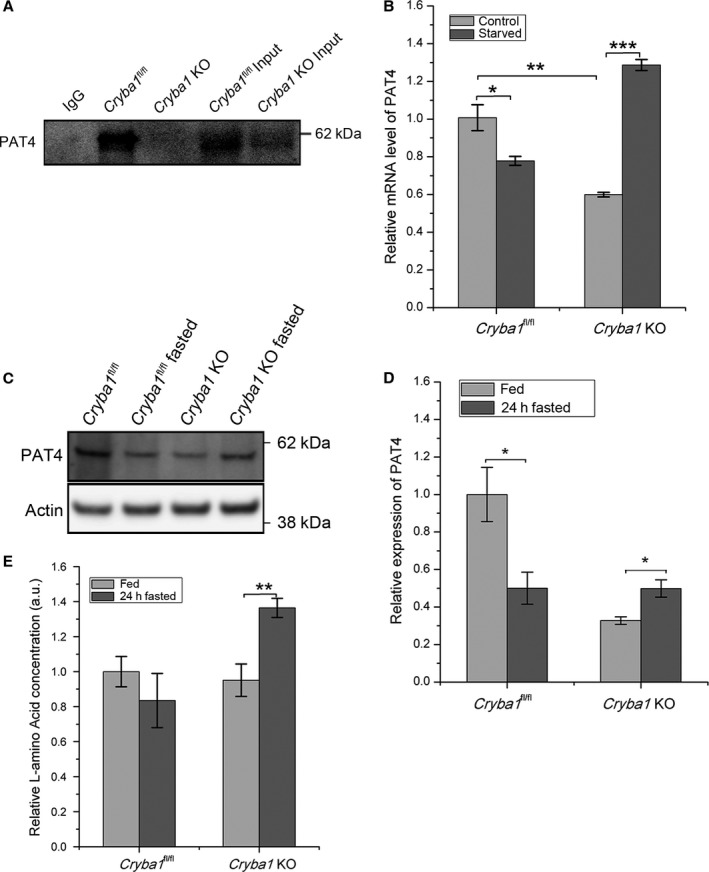
PAT4, interacting with βA3/A1‐crystallin, may modulate the mTORC1 signaling pathway by elevating cellular amino acid concentration. (A) Pull‐down assay using antibody to βA3/A1‐crystallin demonstrates that PAT4 and βA3/A1‐crystallin interact in RPE lysates from two‐month‐old *Cryba1* ^fl/fl^ mice, but not in lysates from *Cryba1* KO mice. (B) qPCR analysis of PAT4 transcript showing lower basal levels in *Cryba1* KO primary RPE cells relative to floxed control samples. Upon serum starvation, the level decreases in RPE cells from floxed mice, but markedly increases in KO cells. (n = 4) (C) Western blot showing corresponding protein levels for PAT4 in RPE (n = 3). Quantification of data in C is shown in D. (E) Total L‐amino acids increase in RPE of *Cryba1* KO mice after 24‐h fasting *in vivo*. No such increase is found in *Cryba1* ^fl/fl^ mice following fasting (n = 8). Actin was used as internal control in all blots. All data expressed as mean ± SEM. *P <* *0.05, **P <* *0.01, ***P <* *0.01.

We recently performed a human proteome high‐throughput array (CDI Laboratories, Inc.) and found that βA3/A1‐crystallin interacts with PAT4/SLC36A4, an amino acid transporter (Supplementary Table 1). The PAT4/SLC36A4 family of amino acid transporters is known to regulate intracellular amino acid concentrations and mTORC1 activity in lysosomes. Here, we show that in a pull‐down assay, βA3/A1‐crystallin binds to PAT4 in RPE cells from two‐month‐old *Cryba1* ^fl/fl^ mice. Such binding did not occur in cells from *Cryba1* KO mice (Fig. 1A). Further, PAT4 RNA (from primary RPE cells in culture) and protein (RPE from tissue) levels were determined by quantitative PCR (QPCR) and western blot, respectively, in RPE of *Cryba1* ^fl/fl^ and *Cryba1* KO mice after fasting. In *Cryba1* ^fl/fl^ (control) mice, PAT4 RNA, as well as protein levels, were downregulated in RPE following 24‐h fasting (Fig. 1B–D). However, in *Cryba1* KO mice, both PAT4 RNA and protein expression increased in the RPE after 24‐h fasting (Fig. 1B–D). *Cryba1* KO mice appear to have a lower basal level of PAT4 expression compared with control mice. PAT4 has previously been shown to regulate amino acid sensing inside cells (Matsui & Fukuda, 2013). Interestingly, our data indicate that the concentration of free L‐amino acids after 24 h of fasting is significantly elevated in RPE cells of *Cryba1* KO mice relative to fed controls. This increase is not seen in cells from *Cryba1* ^fl/fl^ following fasting (Fig. 1E).

**Figure 2:**
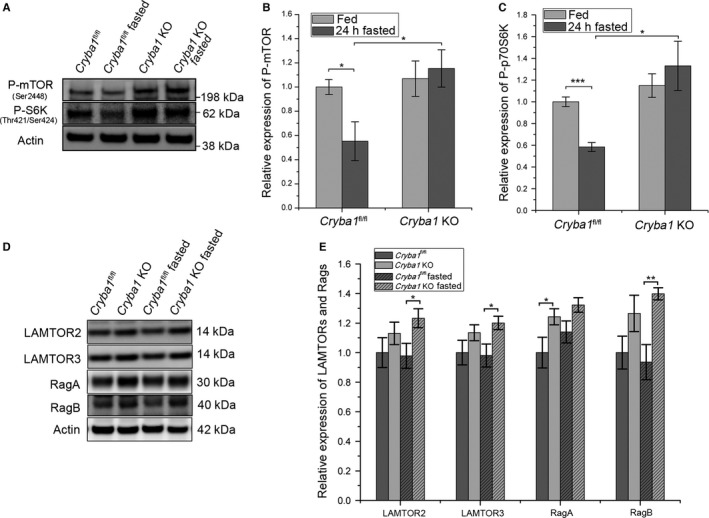
Increased mTORC1 signaling intermediates and persistent activation of mTORC1 signaling pathway in RPE of *Cryba1* KO mice even after fasting. (A) Representative western blot of p‐mTOR (Ser2448) and p70S6K (Thr421/Ser424) in RPE lysates from fed and 24‐h‐fasted *Cryba1* ^fl/fl^ or *Cryba1* KO mice (n = 4). (B) and (C) show densitometric quantification for p‐mTOR and p‐70S6K, respectively. (D) Representative western blots for LAMTOR2, LAMTOR3, RagA, and RagB expression and (E) densitometric quantification of the western blot data for RPE lysates from fed and 24‐h‐fasted *Cryba1* ^fl/fl^ or *Cryba1* KO mice (n = 5). Actin was used as internal control in all blots. Values were normalized to that of fed *Cryba1* ^fl/fl^ samples which relative expression was as 1. All data are expressed as mean ± SEM. *P <* *0.05, **P <* *0.01.

It is known that when the cellular environment is amino acid rich, mTORC1 is activated. When *Cryba1* ^fl/fl^ mice were fasted for 24 h, we found that phosphorylated mTORC1 (Ser2448) and phosphorylated p70S6K (T421/S424) levels decreased in RPE cells, as compared to fed mice. However, phosphorylated mTORC1 (Ser2448) and phosphorylated p70S6K (T421/S424) were observed at consistently higher levels in cells from *Cryba1* KO mice, even after fasting, suggesting activation of mTORC1 (Fig. 2A–C).

It is also true that amino acid‐sensing interactions are required for proper nucleotide loading of the Rag GTPases, recruitment of mTORC1 to the lysosome, and the subsequent activation of mTORC1 (Zoncu *et al*., 2011). The Rag GTPases reside on the lysosome and modulate amino acid import. They exist as obligate heterodimers (RagA or RagB with RagC or RagD) and interact with Ragulator (LAMTOR1‐5). The Ragulator‐Rag multiprotein complex is a critical component in the shuttling of mTORC1 to late endosomes/lysosomes. The protein levels of Ragulator and Rag GTPases, as indicated by western analysis of the mTORC1 signaling intermediates (LAMTOR2, LAMTOR3, RagA, and RagB), were higher in *Cryba1* KO mice than in floxed control mice, but only RagA was statistically significantly higher (Fig. 2D,E). After 24 h of fasting, the levels of LAMTOR2, LAMTOR3, and RagB were statistically significantly higher in cells from KO mice relative to floxed controls.

### Loss of βA3/A1‐crystallin in RPE cells affects TFEB/TFE3 phosphorylation as well as expression of CLEAR network genes

**Figure 3:**
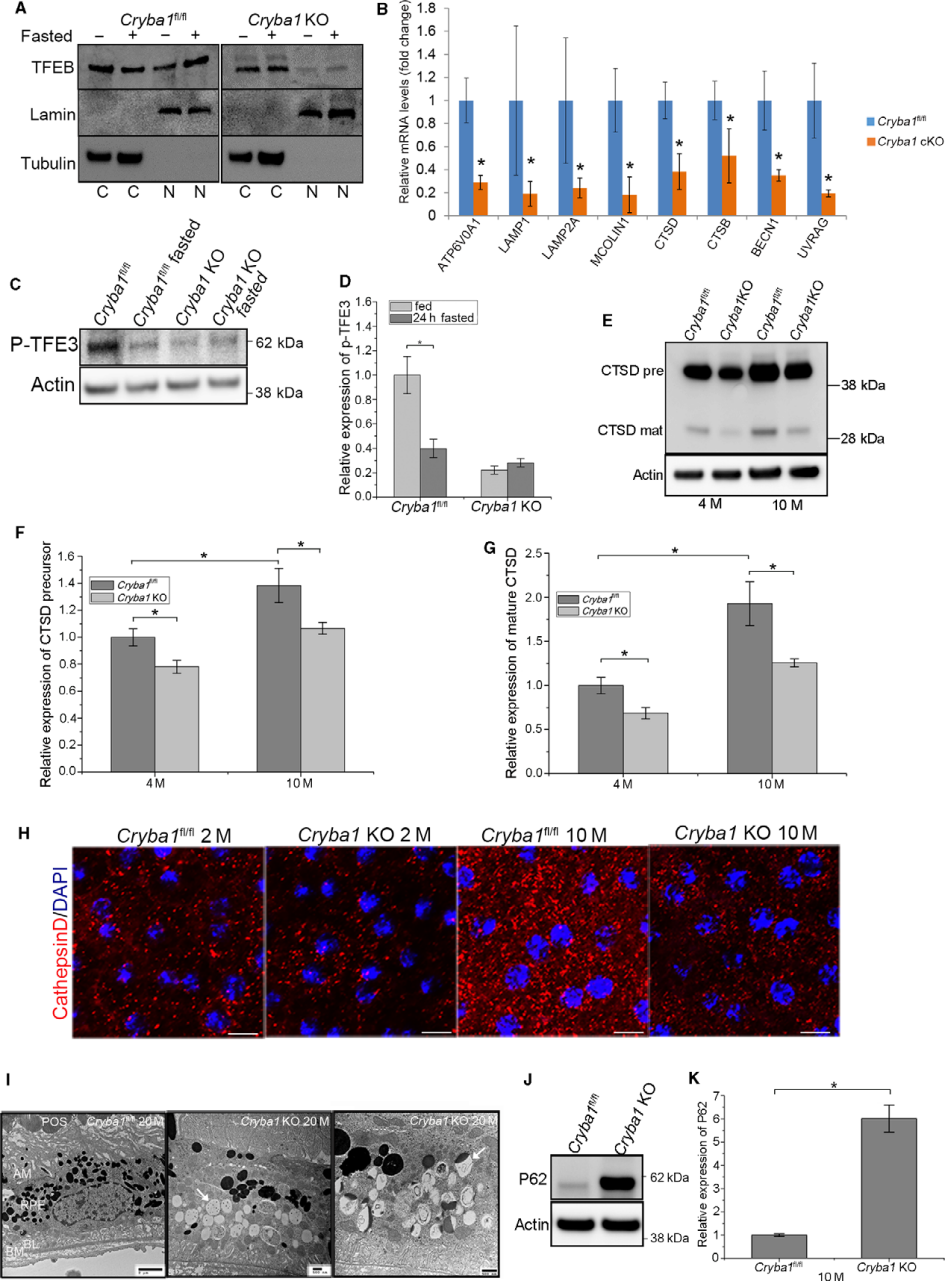
Predominantly cytosolic TFEB and abnormal levels of phosphorylated TFE3 in RPE cells from fasted and fed *Cryba1* KO mice. (A) Western blot showing increased levels of TFEB in *Cryba1* ^fl/fl^ nuclear extracts following fasting. TFEB is predominantly localized in the cytosol (C) in cells from normally fed *Cryba1* ^fl/fl^ (control) mice, but after fasting the nuclear (N) proportion increases. In cells from *Cryba1* KO mice, significantly lower nuclear levels of TFEB were observed in both fasted and fed conditions. (B) *Cryba1* ^fl/fl^ and *Cryba1* KO cells were subjected to qPCR analysis using Taqman probes for some CLEAR (coordinated lysosomal expression and regulation) genes after fasting. *Cryba1* KO cells showed a reduction in the transcript levels for lysosomal hydrolases: CTSD (55%), CTSB (75%); lysosomal acidification: ATP6V0A1 (56%); lysosomal membrane proteins: LAMP1 (15%), LAMP2 (40%), and MCOLIN1 (35%); and gene related to autophagy: BECN1 (51%), UVRAG (35%). The graph shows mean ± SD from triplicate experiments, representative of at least three independent experiments. (two‐tailed t‐test) (C) Western blot for p‐TFE3 in RPE isolated from *Cryba1* ^fl/fl^ and *Cryba1* KO mice. The data indicate decreased p‐TFE3 in controls, but increased p‐TFE3 in KO RPE after fasting. (n = 4) Quantification of C is shown in D. (E) Western blot showing cathepsin D (both precursor and immature forms) expression in the RPE from four‐month‐old (n = 6) and 10‐month‐old (n = 3) *Cryba1* KO mice vs. floxed controls. F and G show densitometric quantification for data in E. (H) CTSD immunostaining (red) and DAPI staining (blue) on RPE flat mounts from *Cryba1* ^fl/fl^ and *Cryba1* KO mice at 2 and 10 months of age (n = 3). There are fewer fluorescent puncta in *Cryba1* KO RPE cells, than in *Cryba1* ^fl/fl^ RPE at both ages. Scale bars=10 μm. (I) TEM of RPE in 20‐month‐old *Cryba1* ^fl/fl^ mouse showing photoreceptor outer segments (POS) and RPE (left panel). Unlike *Cryba1* KO mouse, TEM (center panel) showed numerous vacuoles with possible accumulation of lipid‐like droplets (arrow). TEM also showed many autolysosomes, some with incomplete degradation (arrow) retained in 20‐month‐old *Cryba1* KO mouse (right panel). (J) Western blot and quantification (K) of protein level of SQSTM1 (p62) in RPE of 10‐month‐old *Cryba1* KO mice relative to control mice. (n = 3) *P < 0.05, **P <* *0.01.

mTORC1 modulates the stress‐induced transcription factor EB (TFEB) to regulate a group of genes known as the coordinated lysosomal expression and regulation (CLEAR) network, which maintain normal lysosomal function. Amino acids can regulate TFEB through mTORC1. TFEB, when phosphorylated by mTORC1, is retained in the cytoplasm; when not phosphorylated, it translocates to the nucleus and activates CLEAR genes, thereby stimulating lysosomal biogenesis and function (Settembre *et al*., 2012; Martina *et al*., 2012; Roczniak‐Ferguson *et al*., 2012). Our data suggest that even when autophagy is induced in the RPE by fasting *in vivo*, the absence of βA3/A1‐crystallin causes TFEB to remain in the cytosol, thereby preventing activation of CLEAR genes (Fig. 3A,B). Under normal physiological conditions, western analyses detected TFEB in both the cytoplasm and nucleus of RPE cells of *Cryba1* ^fl/fl^ mice, with an increased proportion in the nucleus after fasting (Fig. 3A). In *Cryba1* KO mouse RPE, TFEB was predominantly cytoplasmic, with no indication of movement into the nucleus after fasting (Fig. 3A). Quantitative real‐time PCR showed that expression levels of lysosomal genes in the CLEAR network were significantly lower in RPE of *Cryba1* KO mice than in controls (Fig. 3B). TFE3, similar to TFEB, is also involved in nutrient sensing and maintenance of cellular homeostasis. TFE3 accumulates in the nucleus upon nutrient deprivation, but is retained in the cytosol when phosphorylated by mTORC1 (Martina *et al*., 2014). In *Cryba1* ^fl/fl^ control mice, phosphorylated TFE3 decreased in RPE after 24‐h fasting, indicating the accumulation of TFE3 in the nucleus. In contrast, the level of phosphorylated TFE3 was not reduced in the RPE of fasted *Cryba1* KO mice like *Cryba1* ^fl/fl^ control mice, even though the phosphorylated TFE3 level in RPE of KO mice is much lower than that in RPE of control mice (Fig. 3C,D).

We further investigated the expression of cathepsin D (CTSD), a CLEAR network gene with significantly decreased expression in *Cryba1* KO RPE relative to control. Interestingly, while CTSD expression increased significantly with age in RPE cells from *Cryba1* ^fl/fl^ mice (Fig. 3E,F,G), in *Cryba1* KO cells, the overall CTSD expression level was lower at both ages relative to controls (Fig. 3E,F,G). Further, CTSD immunolabeling suggests that the capacity of intracellular degradation in the RPE of *Cryba1* KO mice is considerably less than in control mice (Fig. 3H). Our transmission electron microscopy (TEM) data from 20‐month‐old KO mice show accumulation of undegraded material in the RPE (Fig. 3I) as compared to control. Numerous lipidated vacuoles (Fig. 3I, middle and right panels) and, most importantly, greater accumulation of autolysosomes (Fig. 3I, right panel) result from impaired lysosome‐mediated degradation and recycling. We also found that levels of p62, a receptor for cargo destined to be degraded by autophagy, were higher in 10‐month‐old *Cryba1* KO RPE cells than in controls (Fig. 3J,K).

### *Cryba1* deprivation leads to age‐dependent defects in architecture of RPE cells

**Figure 4:**
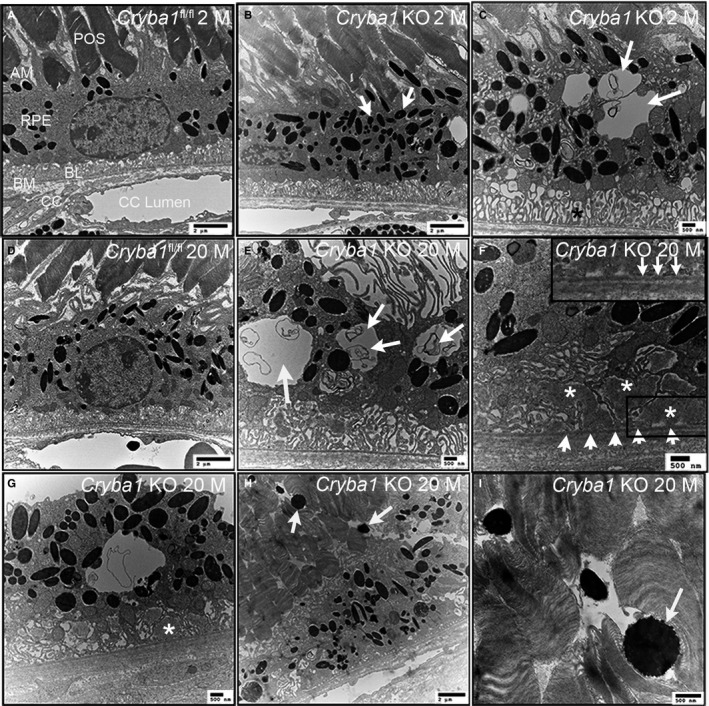
Ultrastructure of RPE in two‐month‐old and 20‐month‐old *Cryba1* knockout mice and age‐matched *Cryba1* ^fl/fl^ mice by transmission electron microscopy. POS (photoreceptor outer segment), AM (apical microvilli), BL (basal lamina), BM (Bruch's membrane), CC (choriocapillaris). (A) RPE of a two‐month‐old *Cryba1* ^fl/fl^ mouse showing normal phenotype. (B) RPE of a two‐month‐old *Cryba1* KO mouse showing increased melanosomes (arrows). (C) Vacuoles appear in RPE of two‐month‐old KO mice (arrows). (D) RPE of a 20‐month‐old *Cryba1* ^fl/fl^ mouse. (E) RPE of a 20‐month‐old *Cryba1* KO mice with much bigger vacuoles containing undigested cellular debris (arrows) and highly disrupted basal infoldings. (F, G) Asterisks indicate basal laminar deposits in KO RPE cells. Arrow heads in F indicate a layer of possible basal laminar deposit between Bruch's membrane and RPE. Inset in F is magnified area in the lower right frame of F, showing a thin part of Bruch's membrane. (H, I) Melanosomes (arrows) from 20‐month‐old KO RPE move into the POS layer.

We next asked whether molecular dysregulation of normal lysosomal function in *Cryba1* KO RPE cells has an effect on RPE structure and, most importantly, whether waste products accumulate in the *Cryba1* KO RPE cells. We observed abnormalities in the cellular architecture of *Cryba1* KO RPE by TEM (Fig. 4). Large vacuoles, not seen in *Cryba1* ^fl/fl^ cells (Fig. 4C), and increased numbers of melanosomes (Fig. 4B) were observed in RPE cells from two‐month‐old *Cryba1* knockout mice, as compared to controls (Fig. 4A). These abnormalities became more severe as the animals aged. Cellular debris enclosed in vacuoles was not efficiently digested (Fig. 4E). In some areas, the *Cryba1* KO RPE cells began to lose basal infoldings and showed intracytoplasmic disruption (Fig. 4F). Such abnormalities were not seen in floxed controls (Fig. 4D). In *Cryba1* KO animals, melanosomes were sometimes found to move from RPE cells into the photoreceptor out segments (POS). Large basal laminar deposits could be seen in *Cryba1* KO RPE (Fig. 4G–I) and were also found above Bruch's membrane (Fig. 4F).

### The visual cycle is impaired in aging *Cryba1* knockout mice

**Figure 5:**
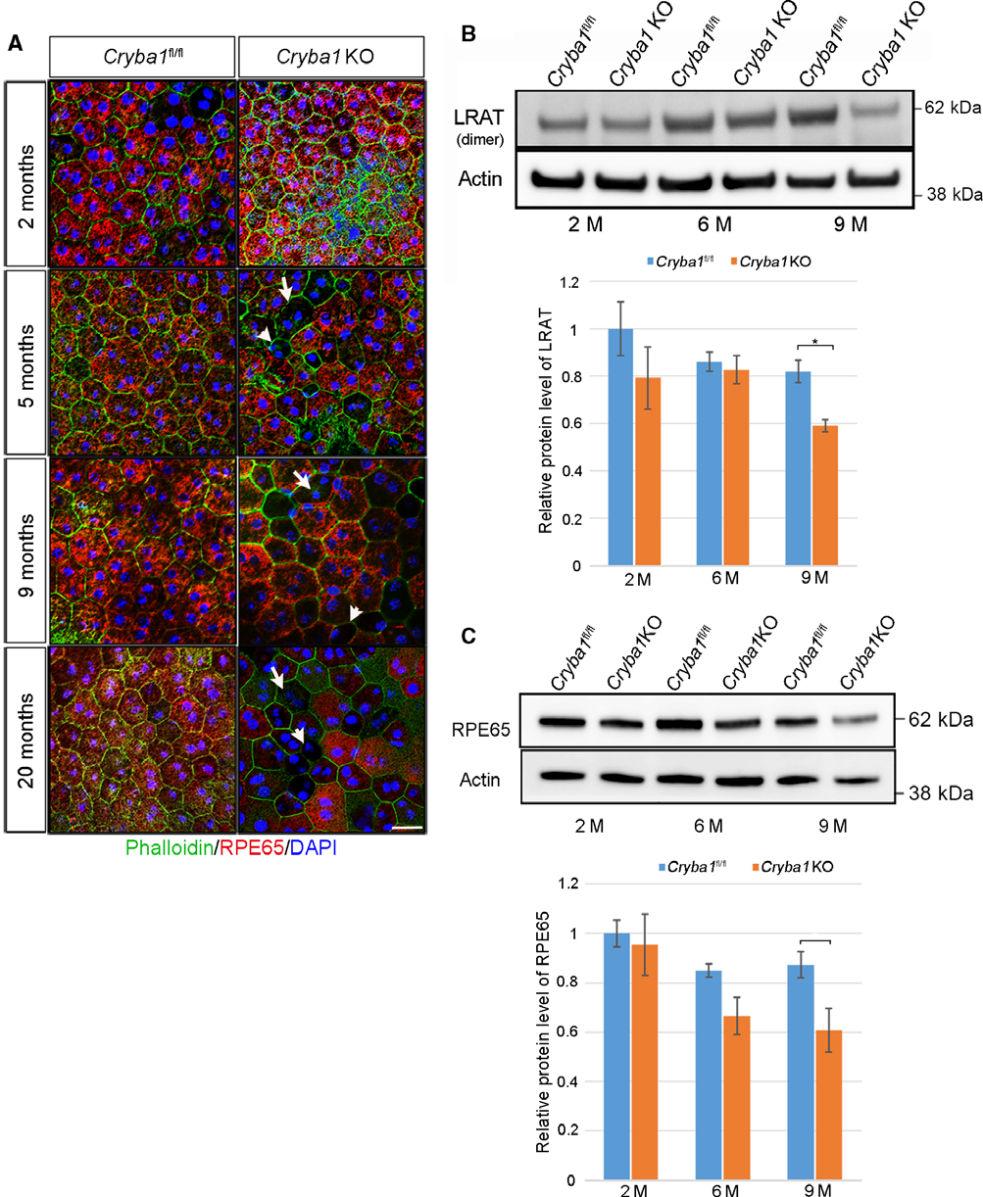
Impaired visual cycle in *Cryba1* KO mice. (A) RPE65 (retinal pigment epithelium‐specific 65 kDa protein, red) and phalloidin (green) immunofluorescent staining of *Cryba1* ^fl/fl^ and *Cryba1* KO mice RPE flat mounts. With increasing age, RPE cells in *Cryba1* KO mice gradually lose their regular hexagonal shape (arrow heads and have less RPE65 (arrows). Scale bar = 20 μm. (B, C) Western blots and densitometric quantification show that as *Cryba1* KO mice age, there is significant loss of RPE65 and LRAT (Lecithin retinol acetyltransferase) in *Cryba1* KO mice by 9 months.

One of the critical functions of RPE cells is the recycling of retinoids that are essential for the visual cycle. RPE65 and lecithin retinol acyltransferase (LRAT) are key enzymes in converting all‐trans‐retinal to 11‐cis‐retinal (Redmond *et al*., 1998; Jin *et al*., 2007). RPE flat mounts from *Cryba1* ^fl/fl^ and *Cryba1* KO mice were stained with the high‐affinity filamentous actin probe, phalloidin, and with RPE65 antibody. Phalloidin staining demonstrated differences in both the size and shape of RPE cells in *Cryba1* KO mice (Fig. 5A). A large number of RPE cells in *Cryba1* KO mice gradually lose their regular hexagonal shape (arrow heads) and exhibit reduced staining for RPE65 (arrows) as they age (Fig. 5A). Both RPE flat mounts and western blots showed a gradual loss of RPE65 in *Cryba1* KO mice as a function of aging. LRAT was also reduced in the *Cryba1* KO mice by 9 months of age compared with age‐matched *Cryba1* ^fl/fl^ mice (Fig. 5B,C).

### Photoreceptor OS dysfunction due to abnormal lysosomal‐mediated clearance of RPE cells from *Cryba1* KO mice

**Figure 6:**
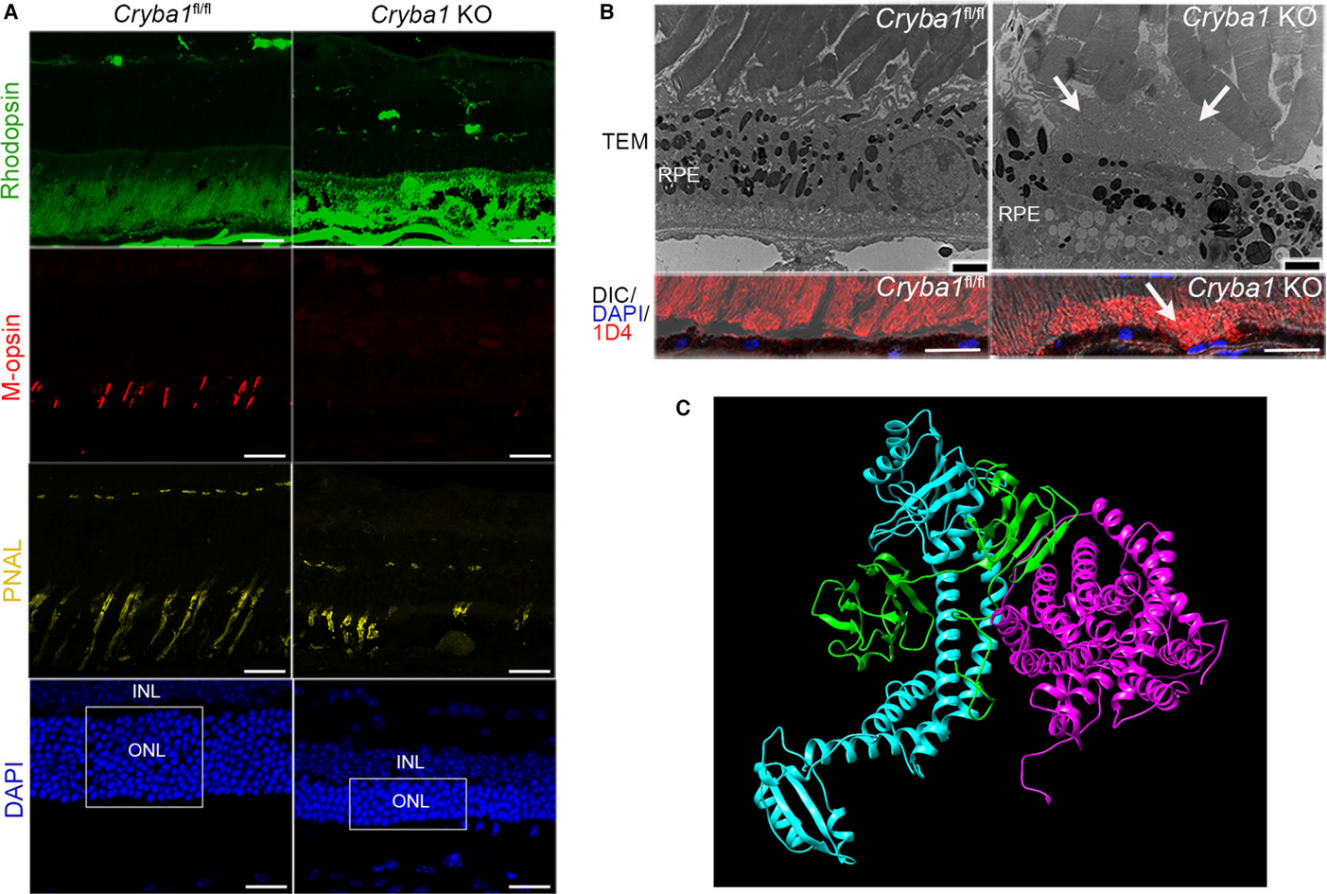
Photoreceptor outer segment dysfunction due to abnormal lysosomal‐mediated clearance of RPE cells from *Cryba1* KO mice. (A) antibodies to rhodopsin (labels rod photoreceptors—green), M‐opsin (labels cones—red), and DAPI (labels nuclei—blue) were used to stain retinal cross sections. Representative data of immunofluorescent staining of photoreceptors: rhodopsin, M‐opsin, peanut agglutinin lectin (PNAL) in 20‐month‐old *Cryba1* KO and age‐matched *Cryba1* ^fl/fl^ retina. DAPI staining showed the number of nuclei and the thickness of outer nuclear layer. ONL (outer nuclear layer), INL (inner nuclear layer), scale bar = 20 μm. (n = 2) (B) Transmission electron microscopy (scale bar = 2 μm) and 1D4 (a marker for shed outer segments) immunostaining (scale bar = 20 μm) showing accumulation of photoreceptor outer segment tips in the subretinal layer in 20‐month‐old *Cryba1* KO mice (arrows), but not in the age‐matched *Cryba1* ^fl/fl^ mice. (C) Three‐dimensional modeling is shown, depicting structures of PAT‐4 (magenta), βA3‐crystallin (green), and V‐ATPase (Cyan) forming a complex, obtained by Hex Protein Docking.

Several elegant studies have suggested that a symbiotic relationship between photoreceptors and RPE cells is necessary for maintaining the proper health of the neural retina (Sparrow *et al*., 2010). As the normal functioning of the RPE is compromised in *Cryba1* KO mice, we evaluated their POS. Immunofluorescence studies for rhodopsin show stronger staining in *Cryba1* KO retinas in cryosections from 20‐month‐old mice compared with those of *Cryba1* ^fl/fl^, but the staining was more diffuse (first panel in Fig. 6A). These data are consistent with staining caused by shed photoreceptor OS, which were not engulfed by RPE cells, but accumulated between the photoreceptors and the RPE. This was confirmed by TEM and 1D4 (POS marker) staining (Fig. 6B). M‐opsin and peanut agglutinin lectin (PNAL), cone photoreceptor markers, were almost undetectable in some regions of the *Cryba1* KO retina (middle two panels in Fig. 6A). Furthermore, the thickness of the outer nuclear layer was significantly reduced in the regions with loss of M‐opsin and PNAL (bottom panel in Fig. 6A), indicating degeneration of photoreceptors in 20‐month‐old *Cryba1* KO mice. TEM also showed degenerating POS in *Cryba1* KO compared with floxed controls.

## Discussion

Many age‐related diseases, including dry AMD, severely impair quality of life in the elderly. We and others have postulated that abnormal lysosome function in RPE cells could ultimately contribute to dry AMD (Guha *et al*., 2014; Sinha *et al*., 2016). In fact, the efficiency of lysosomes in degrading cellular components declines over time, an effect linked both to aging and the development of age‐related diseases (Cuervo & Dice, 2000). Therefore, we have generated unique mouse models to evaluate defective lysosomal degradative function in the RPE and its possible role in AMD.

RPE cells are postmitotic, have high metabolic activity, and are among the most active phagocytic cells in the body (Strauss, 2005). For efficient cellular clearance in the RPE, normal lysosome function through both phagocytosis and autophagy is key (Boya & Codogno, 2013). Lysosomes are no longer regarded as simply a heterogeneous collection of degradative organelles, but also as a platform for signaling pathways (Puertollano, 2014). Our previous work confirmed that βA3/A1‐crystallin regulates mTORC1 signaling, possibly by modulating the assembly/disassembly of the proton pump, V‐ATPase (Valapala *et al*., 2014a). mTORC1 is a platform for a major signaling axis within lysosomes that supports normal lysosomal function and thus cellular homeostasis (Settembre *et al*., 2013).

It is now clear that amino acids and V‐ATPase are absolutely essential for mTORC1 signaling. Two ubiquitously expressed members of the PAT family, PAT1/SLC36A1 and PAT4/SLC36A4, have been shown to affect mTORC1 activity (Heublein *et al*., 2010; Matsui & Fukuda, 2013; Fan *et al*., 2016; Goberdhan *et al*., 2016). We made the unexpected observation that βA3/A1‐crystallin binds to PAT4. This is the first report showing PAT4 expression in the eye. It is known that when the cellular environment is amino acid rich, mTORC1 is active and inhibits autophagy. Our data provide evidence that when βA3/A1‐crystallin is lacking, fasting upregulates PAT4, cellular amino acid levels increase and mTORC1 is activated. Moreover, Rag GTPases, supported by the Ragulator complex (Bar‐Peled *et al*., 2012), modulate amino acid import by lysosomes. Our data clearly show abnormal regulation of Rag GTPases and mTORC1 activity after fasting in *Cryba1* KO RPE cells.

Alternatively, amino acid sensing might occur by an ‘inside‐out’ mechanism with direct coupling between intralysosomal amino acids and activation of mTORC1 leading to subsequent conformational change in the lysosomal V‐ATPase (Zoncu *et al*., 2011). A direct interaction between V‐ATPase and the Rag GEF (guanine nucleotide‐exchange factor) complex was observed. Interestingly, amino acids weaken the interaction between Ragulator and the V‐ATPase V_1_ domain, but have no effect on the interaction with the V_0_ domain. These amino acid‐sensitive interactions were shown to be essential for proper nucleotide loading of the Rag GTPases, recruitment of mTORC1 to the lysosome, and subsequent activation of mTORC1 (Zoncu *et al*., 2011). However, we have previously shown (Valapala *et al*., 2014a) that βA3/A1‐crystallin binds only to the V‐ATPase V_0_ domain, which is responsible for carrying out proton translocation to the endolysosomal compartments and is critical for pH‐dependent processes (Forgac, 2007; Breton & Brown, 2013). It is highly likely that βA3/A1‐crystallin is an upstream regulator of both V‐ATPase and PAT4 and is essential for mTORC1 signaling in RPE cells.

The cellular demand for amino acids is cell and tissue specific. In fact, a direct role for amino acids in mTOR signaling is supported by the well‐documented observation that treatment of cells with protein translation inhibitors (such as cycloheximide), which contribute to increased intracellular concentration of amino acids, can activate mTORC1 and inhibit autophagy even under nutrient deprivation (Beugnet *et al*., 2003; Watanabe‐Asano *et al*., 2014). This is reminiscent of the situation in our *Cryba1* KO mice, suggesting that for normal mTORC1 signaling and thereby maintenance of lysosomal homeostasis in RPE cells, it is important to have a functional βA3/A1‐crystallin‐PAT4‐V‐ATPase complex.

Amino acids also control lysosomal homeostasis through the regulation of TFEB. TFEB localization and inactivation in lysosomes are dependent upon the activation of Rag GTPases and mTORC1 (Martina *et al*., 2012; Settembre *et al*., 2012; Martina & Puertollano, 2013). TFEB binds to the cytosolic chaperone protein 14‐3‐3 when phosphorylated and is retained in the cytoplasm; when not phosphorylated, it translocates to the nucleus and activates CLEAR genes (Settembre *et al*., 2011; Martina *et al*., 2012; Roczniak‐Ferguson *et al*., 2012). Cells adapt to diminished amino acid levels by increasing the lysosomal and autophagic compartments in order to maintain a critical level of metabolites. In *Cryba1* KO RPE cells, the amino acid levels are high, and TFEB is retained in the cytoplasm, even after fasting. In contrast, in RPE cells from control *Cryba1* ^fl/fl^ mice, TFEB translocates to the nucleus and activates CLEAR genes after fasting.

Cathepsin D (CTSD), a CLEAR network gene product involved in phagocytic and autophagic degradation in the RPE (Rakoczy *et al*., 1997; Valapala *et al*., 2014a), is significantly decreased in *Cryba1* KO RPE, relative to control. Previously, we found that the absence of βA3/A1‐crystallin decreased CTSD activity in both astrocytes and RPE cells; however, overexpression of βA3/A1‐crystallin in the KO cells restored normal activity (Valapala *et al*., 2013, 2014a). We also found that p62, a receptor for cargo destined to be degraded by autophagy, was higher in 10‐month‐old *Cryba1* KO RPE cells than in controls; this difference was not found in younger mice. p62 contributes to both amino acid sensing and the regulation of autophagy. It has been shown that p62 is required for maximal mTORC1 activity in response to amino acids. p62 is postulated to promote recruitment of mTORC1 to lysosomes via its interaction with raptor (Duran *et al*., 2011). Taken together, our data indicate that normal lysosomal function is significantly perturbed in *Cryba1* KO RPE cells.

Interestingly, Spatacsin KO mice, which progressively lose cortical motor neurons and Purkinje cells, also have defective lysosomal function (Varga *et al*., 2015). Spatacsin is essential for the reformation of lysosomes from autolysosomes *in vivo*. The loss of lysosomes in these mice preceded neuronal degeneration, a situation analogous to the AMD‐like phenotype in the *Cryba1* KO mouse. In *Cryba1* KO mice, the RPE also loses expression of two vital visual cycle proteins, RPE65 and LRAT. A defective visual cycle would slow down the regeneration of new POS. As these mice age, photoreceptor degeneration also becomes obvious, reminiscent of human dry AMD disease.

Here, we provide a direct link between amino acid availability and mTORC1 signaling during aging in our mouse model. If lysosome‐mediated clearance is perturbed during aging, it could have important implications for age‐related disorders, such as AMD. The ability to sense and appropriately respond to cellular stresses, such as amino acid depletion, is commonly diminished during aging. This could have direct consequences for the onset and progression of aging‐related diseases. For AMD patients, targeting the βA3/A1‐crystallin/PAT4/V‐ATPase complex (Fig. 6C) in the RPE may be an effective means of preventing or delaying the progression of the disease.

## Experimental procedures

### *Cryba1* global knockout mice

βA3/A1‐crystallin conditional knockout (*Cryba1* cKO) mice were generated as previously described (Valapala *et al*., 2014a,b). The controls used in this study are *Cryba1* ^fl/fl^ mice. It is known that germline deletion of floxed alleles may occur when floxed mice are maintained for multiple generations with the Best1‐*Cre* allele, creating a global knockout of the floxed gene. We used this as a strategy to generate *Cryba1* complete knockout (KO) mice. All animal studies were conducted in accordance with the Guide for the Care and Use of Animals (National Academy Press) and were approved by the Animal Care and Use Committee of Johns Hopkins University.

### Isolation and culture of mouse primary RPE cells

Mouse RPE cells were isolated and cultured as previously described (Valapala *et al*., 2014a).

### Autophagy induction

Autophagy was induced in *Cryba1* ^fl/fl^ and KO mice by withholding food for 24 h, but with no restriction on water availability. For *in vitro* starvation, primary cultures of RPE cells from *Cryba1* ^fl/fl^ and KO mice were maintained in growth medium lacking serum and glutamine for 24 h.

### Antibodies

The following antibodies were used in this study: βA3/A1‐crystallin antibody (described previously Zigler *et al*., 2011), Slc36a4 antibody–N‐terminal (Aviva System Biology, San Diego, CA, ARP44114‐P050), Actin (Sigma, St. Louis, MO, A2066), PhosphoPlus‐p70 S6 Kinase (Thr389, Thr421/Ser424) Antibody Kit (Cell Signaling, Danver, MA, #9430), Phospho‐mTOR (Ser2448) (Cell Signaling, #5536), Rag and LAMTOR Antibody Sampler Kit (Cell Signaling, #8665), TFEB (Bethyl Laboratories, Montgomery, MA, A303‐673A), Lamin A/B (Santa Cruz, sc‐6215), Tubulin (MBL, PM054‐7Y), Phospho‐TFE3 (as described in Martina *et al*., 2016), CTSD (Gift from Dr. Ralph Nixon, NYU School of Medicine), SQSTM1/P62 (Abcam, Cambridge, MA, ab91526), Rhodopsin, M‐opsin, and PNAL (three gifts from Dr. Donald Zack, Wilmer Eye Institute, The Johns Hopkins University School of Medicine), 1D4 (gift from Dr. Krzysztof Palczewski, Case Western University), RPE65 (gift from Dr. T. Michael Redmond, NEI, NIH), and LRAT (Santa Cruz, Dallas, TX, sc‐99015).

### Co‐immunoprecipitation

Pierce Co‐Immunoprecipitation Kit (Thermo Scientific, Waltham, MA, #26149) was used to carry out the immunoprecipitation studies. Briefly, RPE‐choroid preparations from seven mice of each genotype were sonicated in IP Lysis/Wash Buffer (provided in the kit) plus 1% protease inhibitors (Sigma). The total lysates were processed with the kit according to the instructions. Seventy micrograms of lysates of each genotype were immunoprecipitated with 10ug immobilized βA3/A1‐crystallin antibody at 4°C overnight. Normal rabbit IgG (Santa Cruz, sc‐2027) was the negative control. Samples from elution were loaded for SDS‐PAGE analysis. Fifteen micrograms of RPE‐choroid lysates of each genotype were loaded as the input for the SDS‐PAGE analysis.

### Protein extraction and western blot analysis

RPE‐choroid preparations from freshly dissected mice were sonicated in RIPA lysis buffer (Millipore, Billerica, MA, 20‐188) plus 1% protease and phosphatase inhibitors (Sigma). Samples were incubated on ice for 20 min and centrifuged at 13 000 *g* for 20 min. The supernatants were mixed with 4X protein sample buffer (Invitrogen, Carlsbad, CA) plus 5% 2‐mercaptoethanol (Sigma) and heated at 100°C for 10 min to denature. Samples were loaded into a 4–12% Bis‐Tris Nu‐PAGE gel (Invitrogen) and run with MES buffer (Novex, Waltham, MA). Proteins were transferred to nitrocellulose membranes which were then blocked in 5% skim milk (Invitrogen) or 5% BSA (Sigma, for phosphorylated proteins). The membranes were incubated with primary antibody overnight followed by horseradish peroxidase‐conjugated secondary antibodies for 1 h at room temperature. Blots were developed by chemiluminescence (ECL) methodology. Densitometric analysis was carried out using quantity one software (Bio‐Rad Laboratories, Hercules, CA).

### Human proteome high‐throughput array

The human proteome microarray 2.0 analysis was performed as a paid service from CDI NextGen Proteomics, MD, USA. For hit identification, we first obtained the ratio of median value of the foreground to the median of the surrounding background for each protein probe on the microarray, followed by the normalization by the median value of all neighboring probes within a 9 × 9 window size. Then, we compared normalized value of each probe to the distribution of noise signals to obtain a *Z*‐score representing the significance of the probe binding signal different from random noises we chose. The cutoff of *Z*‐score was 6 in this study. The protein was determined as a hit only if its *Z*‐score was above the cutoff for all triplicates.

### Nuclei and cytoplasmic fraction

Nuclear and cytoplasmic fractions were isolated from extract of RPE‐choroid preparations from six‐month‐old *Cryba1* ^fl/fl^ or *Cryba1* KO mice using the NE‐PER Nuclear and Cytoplasmic Extraction Reagent Kit (Thermo Fisher Scientific, Waltham, MA, #78833) according to the manufacturer's protocol.

### Quantification of the intracellular L‐amino‐acid concentration

RPE‐choroid complexes were homogenized in L‐amino acid assay buffer from the L‐Amino Acid Quantification Kit (Sigma, MAK002). Lysates were prepared as described above and then analyzed according to the manufacturer's instructions to determine their intracellular L‐amino‐acid concentration. The value for each sample was normalized by total protein concentration.

### Immunofluorescence of RPE flat mount and cryosections

Fresh eyes were enucleated and fixed in 2% paraformaldehyde (PFA) for 10 min and then the anterior parts including cornea, lens, and attached iris pigmented epithelium were removed. The resulting posterior eyecups were fixed in 2% PFA for 1 h at room temperature either for cryosections or RPE flat mount. For cryosections, the eyecups were dehydrated through gradient sucrose solutions and embedded in OCT. For RPE flat mounts, retinas were then removed after the eyecup was quartered like a petaloid structure. The resulting eyecup was further cut radially into eight pieces from the optic nerve head to the periphery. Immunostaining on flat mount and cryosections was performed as described previously (Zigler *et al*., 2011). Stained RPE‐choroid sheets with sclera were mounted on a microscope slide with RPE layer up. Images were acquired by Zeiss LSM 710 confocal workstation.

### Transmission electron microscopy

Samples were prepared and transmission electron microscopy was performed as previously described (Zigler *et al*., 2011).

### RNA isolation, cDNA synthesis, and qRT–PCR

RNA isolation, cDNA synthesis, and qRT–PCR were performed as described previously (Valapala *et al*., 2014a). PCR amplification was performed using the 7500 PCR Fast Real‐Time System (Applied Biosystems, Carlsbad, CA, USA) and custom‐made TaqMan probes for LAMP1 (Mm00495262_m1), LAMP2A (Mm00495274_m1), cathepsin B (Mm01310506_m1), cathepsin D (Mm00515586_m1), V‐ATPase (Mm00724370_m1), UVRAG (Mm00724370_m1), Beclin‐1 (Mm01265461_m1), and Mucolipin‐1 (Mm00522549_m1). Actin B (Mm00607939_s1) was used as a loading control. All data were analyzed with the ABI 7500 Real Time PCR system using the data assist Software (Applied Biosystems), and the graphs were plotted using microsoft excel, Redmond, WA.

### Molecular modeling

The possible interaction between PAT‐4, βA3‐crystallin, and V‐ATPase was tested using an interactive protein docking and molecular superposition program hex protein docking, version 6.3 (http://hex.loria.fr/manual63/hex_manual.pdf).

### Statistical analysis

Statistical analysis was performed using microsoft excel. Graphs were plotted using origin8 software or microsoft excel. The *P*‐values were determined by two‐tailed Student's t‐test in at least three biological replicate experiments. Significance was defined as *P < 0.05. Results are presented as mean ± SEM.

## Funding

This study was funded by an unrestricted grant to the Wilmer Eye Institute from the Research to Prevent Blindness, National Eye Institute: EY019037‐S (DS), EY019044 (JTH), EY14005 (JTH), EY01765 (Wilmer Imaging Core), and the National High Technology Research and Development Program of China (2013CB967501, 2015CB964601, 2013CB967101), Shanghai East Hospital (ZJ2014‐2D‐002), and Tongji Eye Institute (TEI‐201403001).

## Conflict of interest

None declared.

## Author contributions

DS designed the study and assisted with the generation of *Cryba1* knockout (KO) mice and data analysis. G‐TX participated in the study design and analyzed data. SZ generated the *Cryba1* KO mice and analyzed data. PS conducted the majority of experiments. MV, SG, and RP were involved with TFEB, TFE3, and CLEAR gene network studies. TEM experiments were conducted by RG and analyzed data with JTH, IAB, and JL. SH constructed the figures. JW and JQ analyzed the human proteome high‐throughput array data. LL assisted with the morphological studies and analyzed data. YS did the molecular modeling. DS, SZ, SH, and PS wrote the paper. All authors have approved the final manuscript.

## Supporting information


**Table S1** Heatmap of Z‐scores of 78 protein hits identified for all triplicates (Rep1/2/3) from 14 693 human proteins on the microarray. The hits were sorted by their mean value of Z‐scores.
