# Supplementary figures and images for: The amino acid transporter SLC36A4 regulates the amino acid pool in retinal pigmented epithelial cells and mediates the mechanistic target of rapamycin, complex 1 signaling
Source: Aging Cell. 2017 Jan 13;16(2):349–59. doi: 10.1111/acel.12561 (PMC5334531; doi:10.1111/acel.12561)

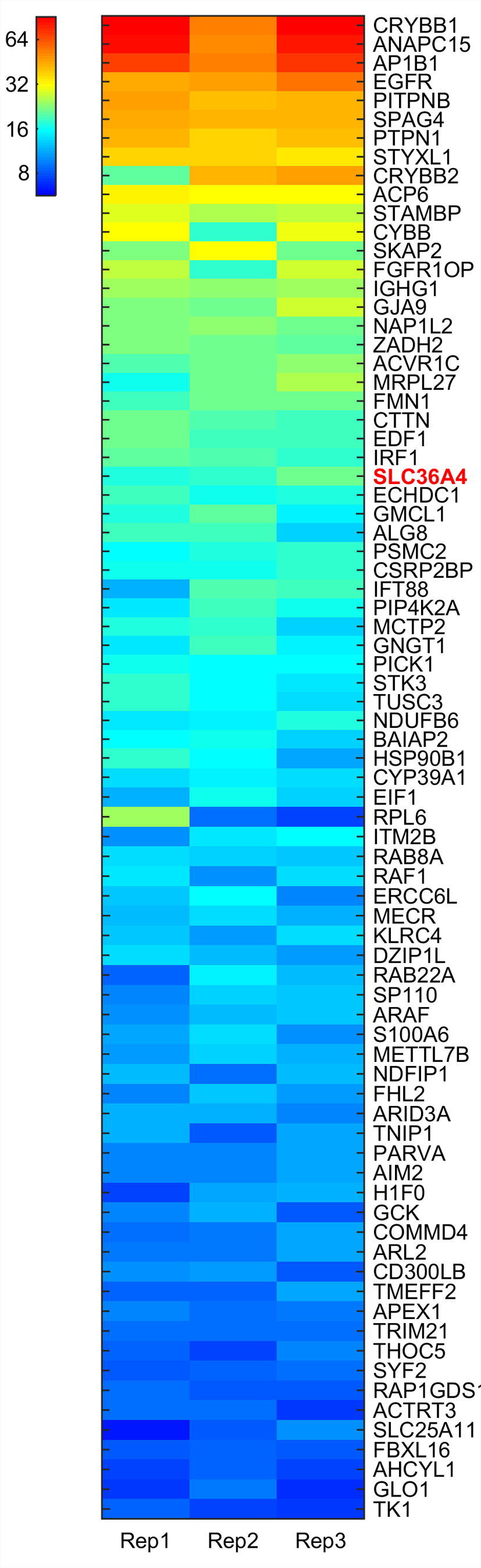

Supplement: Supplementary file 1 — Table S1 Heatmap of Z‐scores of 78 protein hits identified for all triplicates (Rep1/2/3) from 14 693 human proteins on the microarray. The hits were sorted by their mean value of Z‐scores. [file ACEL-16-349-s001.tif]
